# Supplementary material for: A Novel HDL-Mimetic Peptide HM-10/10 Protects RPE and Photoreceptors in Murine Models of Retinal Degeneration
Source: Int J Mol Sci. 2019 Sep 27;20(19):4807. doi: 10.3390/ijms20194807 (PMC6801888; doi:10.3390/ijms20194807)
Supplement: Supplementary file 1 [file ijms-20-04807-s001.pdf]

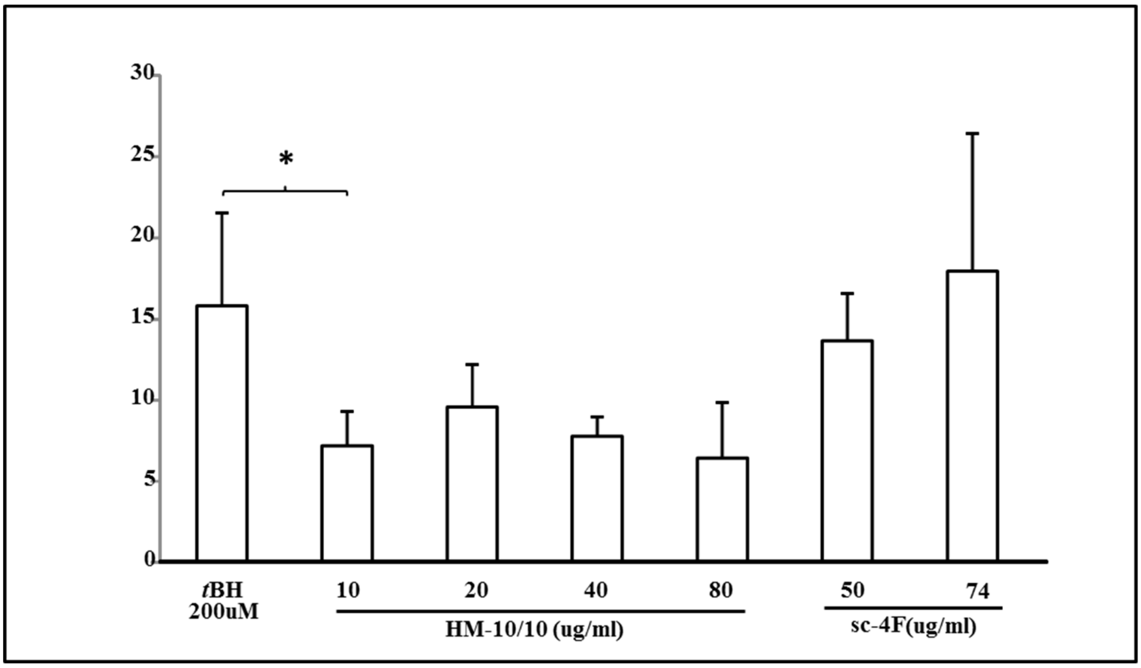

Supplemental Figure S1. Establishment of effective HM-10/10 dosing in hfRPE cells. hfRPE cells were incubated with HM-10/10 at 10, 20, 40, and 80 ug/mL or sc-4F at 50 or 74 ug/mL for 24 h as described under Materials and Methods. Apoptosis was analyzed and quantified by TUNEL staining. Asterisk indicates $p < 0.0024$ with the Bonferroni correction.

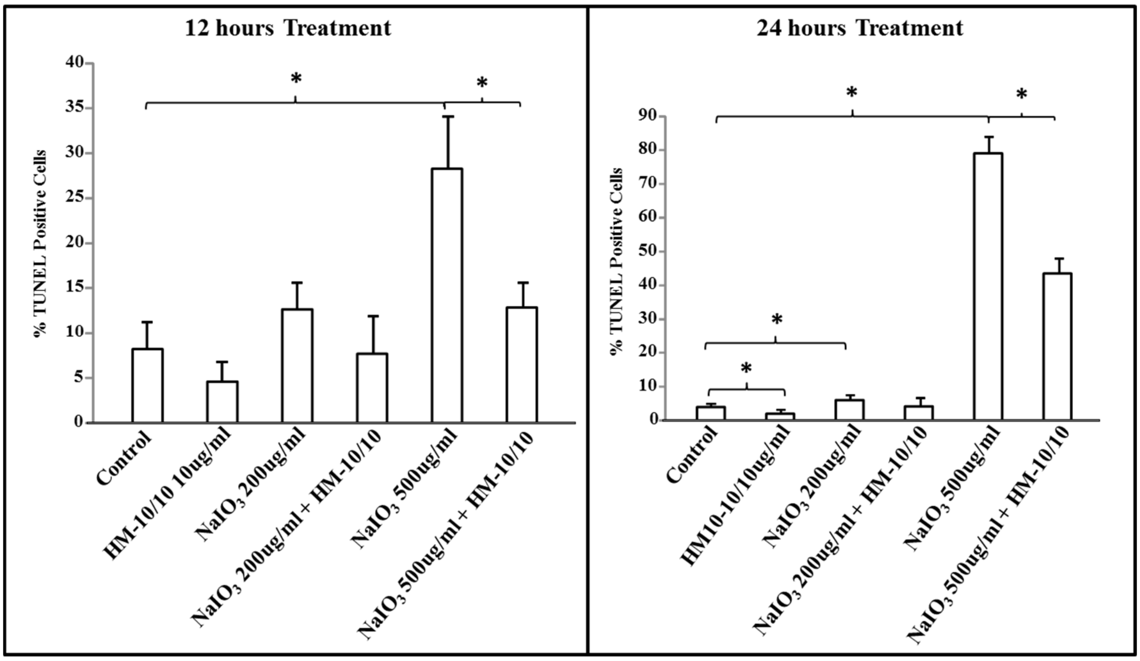

Supplemental Figure S2. HM-10/10 peptide mitigates $NaIO_3$-induced apoptosis of hfRPE cells. hfRPE cells were incubated with HM-10/10 peptide at 10 ug/mL, or $NaIO_3$ at either 200ug/mL or 500ug/mL, or treated with $NaIO_3$ and HM-10/10 together, for 12 h or 24 h as described under Materials and Methods. Apoptosis was analyzed and quantified by TUNEL staining. Asterisk indicates $p < 0.0036$ with the Bonferroni correction.

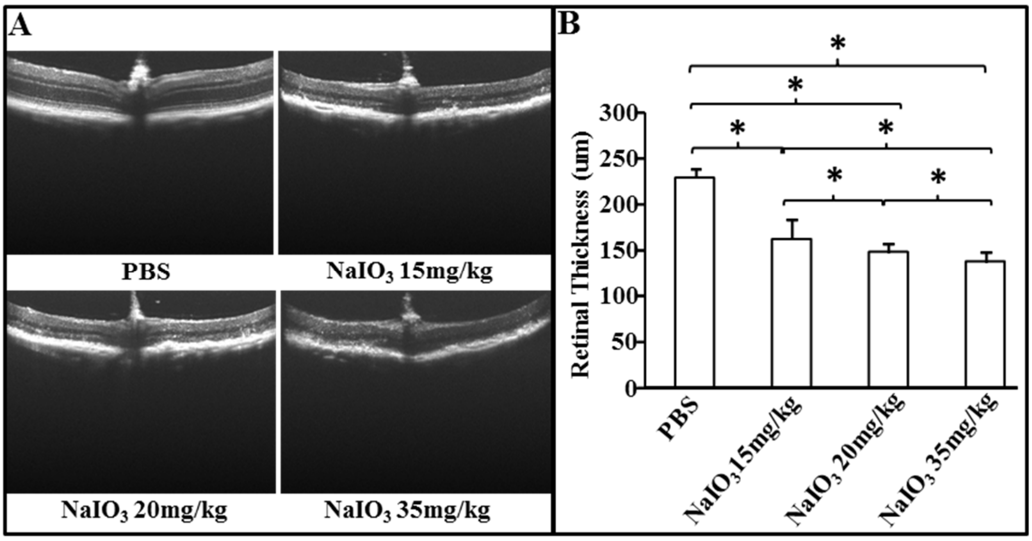

Supplemental Figure S3. Establishment of effective $NaIO_3$ dosing in C57BL6/J mice. C57BL6/J male mice were injected with different doses (as shown) of $NaIO_3$ via tail vein. After a week on a standard chow diet retinal damage was analyzed. (**A**) Representative OCT images. (**B**) Retinal thickness measured as described under Materials and Methods. Asterisk indicates $p < 0.0083$ with the Bonferroni correction.
